# The Unicellular Red Alga *Cyanidioschyzon merolae*, an Excellent Model Organism for Elucidating Fundamental Molecular Mechanisms and Their Applications in Biofuel Production

**DOI:** 10.3390/plants10061218

**Published:** 2021-06-15

**Authors:** Imran Pancha, Kazuhiro Takaya, Kan Tanaka, Sousuke Imamura

**Affiliations:** 1 Department of Biological Sciences, SRM University-AP, Amaravati, Andhra Pradesh 522502, India; 2 NTT Space Environment and Energy Laboratories, Nippon Telegraph and Telephone Corporation, 3-9-11 Midori-cho, Musashino-shi, Tokyo 180-8585, Japan; kazuhiro.takaya.bm@hco.ntt.co.jp; 3 Laboratory for Chemistry and Life Science, Institute of Innovative Research, Tokyo Institute of Technology, 4259-R1-29 Nagatsuta, Midori-ku, Yokohama-shi, Kanagawa 226-8503, Japan; kntanaka@res.titech.ac.jp

**Keywords:** *C. merolae*, metabolic engineering, starch, TAGs, target of rapamycin

## Abstract

Microalgae are considered one of the best resources for the production of biofuels and industrially important compounds. Various models have been developed to understand the fundamental mechanism underlying the accumulation of triacylglycerols (TAGs)/starch and to enhance its content in cells. Among various algae, the red alga *Cyanidioschyzon* *merolae* has been considered an excellent model system to understand the fundamental mechanisms behind the accumulation of TAG/starch in the microalga, as it has a smaller genome size and various biotechnological methods are available for it. Furthermore, *C. merolae* can grow and survive under high temperature (40 °C) and low pH (2–3) conditions, where most other organisms would die, thus making it a choice alga for large-scale production. Investigations using this alga has revealed that the target of rapamycin (TOR) kinase is involved in the accumulation of carbon-reserved molecules, TAGs, and starch. Furthermore, detailed molecular mechanisms of the role of TOR in controlling the accumulation of TAGs and starch were uncovered via omics analyses. Based on these findings, genetic engineering of the key gene and proteins resulted in a drastic increment of the amount of TAGs and starch. In addition to these studies, other trials that attempted to achieve the TAG increment in *C. merolae* have been summarized in this article.

## 1. Introduction

The significant challenges in the 21st century include the increasing human population, decline in fossil energy reserves, global warming, and other environmental issues. Primary research in modern times is devoted to developing sustainable and renewable biofuel production. Microalgae utilize atmospheric CO_2_ and thereby accumulate triacylglycerols (TAGs) and starch in cells, which can act as veritable biofactories for biofuel production [1]. Microalgae have simpler growth requirements and a higher growth rate and photosynthetic ability than land plants. Additionally, microalgae can be cultivated using seawater or wastewater, which addresses the debate of food versus fuel [1]. The challenges of commercializing microalgae-based biofuel include the relatively lower biomass productivity and high costs associated with downstream processing. Various biochemical and genetic engineering approaches have been used to improve the biomass production of microalgae. However, it is unclear how these tiny microalgae accumulate energy reserve compounds. Understanding the molecular mechanism underlying the accumulation of TAGs and starch in microalgae is an important parameter to improve biomass productivity and reduce overall production costs.

Different groups of model unicellular microalgae, such as *Chlamydomonas reinhardtii*, *Nannochloropsis oceanica*, *Phaeodactylum tricornutum*, and *Cyanidioschyzon merolae*, etc., have been used to investigate TAG and starch metabolism [2,3,4]. Recently, using this red alga model, several fundamental molecular mechanisms behind TAG and starch accumulation were revealed, which indicates this might be a good model system to understand TAG and starch biosynthesis in microalgae [5,6,7]. *C. merolae* is an extremophilic red alga that is generally cultivated in a medium with a pH of 2–3 and growth temperature of 40–50 °C [8]. Compared with other model systems, these characteristics make this alga unique, as it can be cultivated in countries in the tropical region where it is difficult for other algae to survive. Additionally, growth at a low pH and high temperature prevents the growth of other organisms and eliminates the possibility of contamination and production loss due to predators. A recent study revealed that a preculture of *C. merolae* in moderate salt concentration further enhanced the ability of the alga to grow in seawater without the addition of buffering agents [9]. Cultivation of this alga in seawater, which is abundantly available as a cheap water resource, would lower production costs and minimize the use of precious limited freshwater. All these growth characteristics of *C. merolae* make it an excellent model system for developing large-scale algal biomass production.

In genetic engineering, *C. merolae* is also a superior model system because of its various characteristics; for instance, it contains a single mitochondrion, single chloroplast, and single nucleus. Moreover, the complete genome sequence of this organelle is freely and publicly available [10,11,12]. Additionally, various genetic and biochemical techniques have been well established for *C. merolae* for studying its various processes, such as transient gene expression [13,14], genome tagging using homologous recombination [15,16,17], overexpression of the transgene [18,19,20], and marker recycling [21]. The establishment of the above methods elucidated the molecular mechanism underlying the accumulation of TAGs and starches in *C. merolae*, which then helped in the development of *C. merolae* as a veritable biofactory for the production of biofuels and other important industry-dependent chemicals. Due to these advantages, *C. merolae* is an excellent model organism to understand the metabolism of TAGs and starch and exploit these capabilities as biofactories for future large-scale biofuel production.

Most microalgae accumulate carbon storage compounds, such as TAGs and starches, under unfavorable growth conditions such as nitrogen depletion [22,23]. However, such stress conditions generally result in lower biomass production, which is not advisable for large-scale microalgal biofuel production. Identification of target genes, transcription factors, or signaling pathway(s) involved in the accumulation of TAGs under stress conditions is important. Genetic engineering of such targets under normal conditions improves the biofuel production capabilities of microalga without compromising its growth. Here, we present an overview of the various molecular approaches, such as identifying important regulators and signaling pathway(s) that regulate TAGs and starch accumulation in *C. merolae*. We also include examples of successful case studies that have genetically generated and manipulated *C. merolae* strains to accumulate higher amounts of TAGs and/or starch in cells via genetic engineering.

## 2. Metabolic Engineering of *C. merolae* for Biofuel Production

Metabolic pathways and enzymes that are involved in the synthesis of fatty acid and TAG in *C. merolae* have been well documented. These processes in alga are similar to those in land plants apart from a few key differences [24]. Similar to land plants, fatty acids such as C16:0 and C18:0 are synthesized in plastids in *C. merolae*, and further chain-length elongation and desaturation occurs in the endoplasmic reticulum (ER). However, an important characteristic of the *C. merolae* fatty acid synthesis pathway is that it lacks plastidial desaturases [24]. Additionally, the unsaturated fatty acids are components of chloroplast in *C. merolae* similar to the other algae and plants. These indicate they have couple pathways to synthesize plastidial lipids such as monogalactosyldiacylglycerol (MGDG). MGDG is synthesized with C18:2, while C18:2 cannot be supplied by plastids, as *C. merolae* do not have desaturase in plastids. Therefore, C18:2 must be supplied from the extraplastidic (e.g., ER), which is interesting characteristic of the couple pathway [24]. As mentioned earlier, microalgae accumulate TAGs during various stress conditions, such as nitrogen depletion, which results in lower biomass and makes this method unsuitable for commercial-scale TAG production. To overcome the limitation of these cultivation-based approaches, metabolic engineering is a promising way to enhance the TAG content in cells without affecting normal growth and biomass production. One of the primary targets for metabolic engineering in microalgae for biofuel production is to reduce the degradation of accumulated TAGs in the cells or to increase the substrate for fatty acid synthesis and/or TAG synthesis [25]. However, an earlier attempt to create a genetically modified strain of diatoms with overexpression of acetyl-CoA carboxylase (ACCase) did not significantly increase TAG content [26]. Careful selection of a gene or target is essential in genetic engineering and the successful production of energy-reserved compounds in target algae.

**Figure 1:**
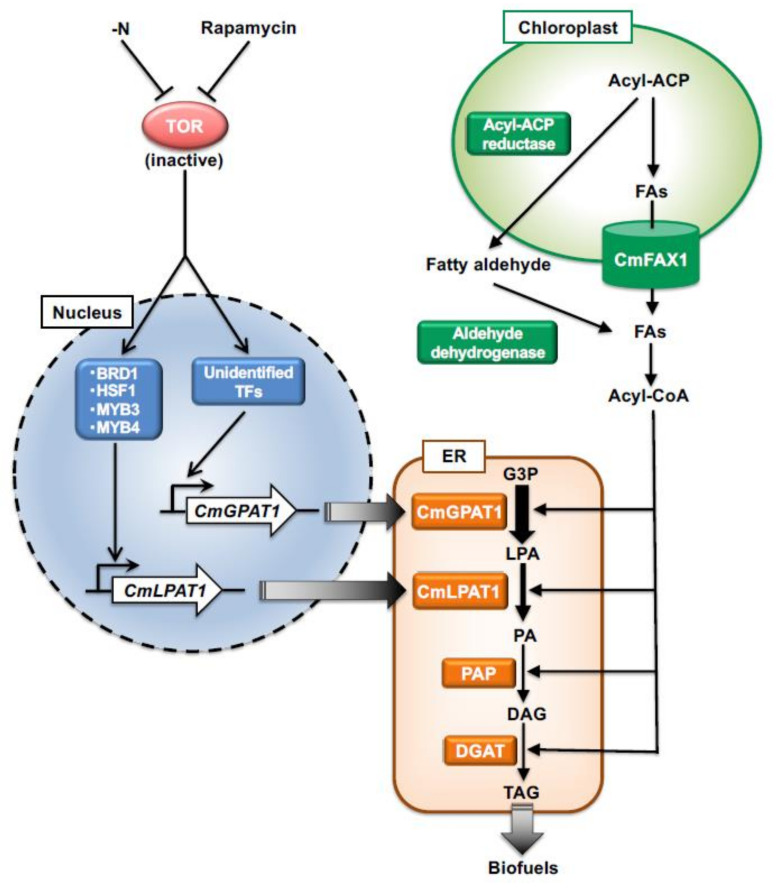
The metabolic engineering strategies to improve TAG content in *Cyanidioschyzon merolae*. Under the target of rapamycin (TOR), inactivation conditions by rapamycin resulted in TAG accumulation similar to the nitrogen depletion, indicating TOR is the checkpoint kinase that determines TAG accumulation in the cells. The TOR function on the TAG accumulation is conserved among plant lineages as general. Under the TOR inactivation or nitrogen depletion conditions, the transcript levels of *CmGPAT1* and *CmLPAT1* are upregulated significantly. Although four transcription factors (TFs) are identified for the regulation of *CmLPAT1* transcription, TF(s) for *CmGPAT1* transcription is not known yet. Overexpression of CmGPAT1 drastically increases the amount of TAGs, whose amounts are almost similar to the nitrogen depletion condition in the cells under the normal growth condition. When CmLPAT1 is overexpressed, the amount of TAGs is significantly increased. This indicates the steps catalyzed by CmGPAT1 and CmLPAT1 are important for TAG biosynthesis with different contributions in *C. merolae*. Overexpression of CmFAX1 or heterologous expression of cyanobacterial Acyl-ACP reductase in *C. merolae* cells also increases the TAG content in cells. See the details in the text.

In the very first attempt at metabolic engineering of *C. merolae*, Sumiya et al. (2015) expressed Acyl–Acyl carrier protein (acyl-ACP) reductase from cyanobacteria to enhance TAG productivity in this alga (Figure 1). The authors used this strategy because the overexpression of acyl-ACP reductase reportedly resulted in higher fatty acid accumulation in cyanobacteria [27]. However, this overexpression alone is not sufficient for promoting the increased accumulation of TAG; it requires additional oxidation of fatty aldehyde through an aldehyde dehydrogenase. *C. merolae* possess aldehyde dehydrogenase (CMO345C in *C. merolae* genome database http://czon.jp, accessed on 10 March 2021) but not acyl-ACP reductase. Therefore, the expression of cyanobacterial acyl-ACP reductase in chloroplast might improve the TAG content by increasing the fatty acid content in *C. merolae*. To construct a genetically modified *C. merolae* strain, the acyl-ACP reductase gene from cyanobacterium *Synechocystis* sp. PCC 6803 was inserted into the genome of *C. merolae* [28]. The resulting genetically modified strain was named AAR-3HA. Neutral lipid accumulation characteristics of the AAR-3HA strain were observed using the BODIPY staining method, which indicated an accumulation of clear, high-lipid droplets that deviated from the characteristics of the control strains [28]. Furthermore, quantification of TAGs using a gas chromatography flame ionization detector (GC-FID) indicated an accumulation of TAGs in the AAR-3HA strain that was approximately three times higher than that in the control without acyl-ACP reductase expression. The study also confirmed the essential role of the *C. merolae* aldehyde dehydrogenase (CMO345C). The expression of cyanobacterial acyl-ACP reductase in CMO345C knockout background did not show any significant difference in the TAG levels [28].

To further identify how the expression of acyl-ACP reductase results in TAG accumulation in *C. merolae*, the authors conducted gene expression analysis and capillary electrophoresis mass spectrometry-based metabolome analysis, and the results indicated that the acyl-ACP reductase expression upregulated several vital steps and metabolic pathways in the accumulation of TAGs within cells. For example, the AAR-3HA strain exhibited significantly higher expression of chloroplast heteromeric ACCase only after 1 day of cultivation; similarly, the activity of ACCase in the cell extract of AAR-3HA was 4.4 times higher than in that of the control strain [28]. Additionally, the results of metabolomics and transcriptomic indicated a different route for the accumulation of TAGs via the overexpression of acyl-ACP reductase than nitrogen depletion conditions in *C. merolae*. Generally, under nitrogen depletion conditions, the expression levels of genes involved in the pentose-phosphate pathway are upregulated, but in AAR-3HA cells, the expression levels of genes such as G6PD were downregulated, and those of chloroplast ACCase were upregulated, indicating that an additional supply of carbon source does not result from the degradation of chlorophyll and proteins [28]. Cumulatively, these results indicate that heterologous expression of the cyanobacterial gene in *C. merolae* markedly improves TAG content and its subsequent application in biofuel production. Further studies on the expressions of other genes from cyanobacteria and other oleaginous organisms might lead to improvements in the biomass production of this alga. Such metabolic engineering strategies are important for the production of sustainable biofuels and valuable compounds from *C. merolae*.

## 3. Role of TOR Signaling in TAG Accumulation

Target of rapamycin (TOR) is the checkpoint kinase that regulates growth, development, and cellular energy metabolism in eukaryotes [29]. In eukaryotes, conserved serine/threonine kinase TORs sense the nutrient and energy statuses and influence metabolic change in response to such external inputs [29]. Additionally, nitrogen depletion causes an effect in metabolism that is similar to TOR inactivation [30,31]; this indicates that TOR might be involved in TAG accumulation in microalgae. In yeast and mammals, macrolide antibiotic rapamycin binds with a small 12 kDa protein, known as the FK506-binding protein (FKBP12), and inhibits kinase activity of TOR [32,33,34]. Recently, it was found that TOR inactivation through rapamycin results in lipid droplet accumulation in *Saccharomyces cerevisiae* [35]. Additionally, it has been reported that TOR inactivation results in TAG accumulation in the model plant *Arabidopsis thaliana* [36]. Such results suggest that TOR is involved in TAG regulation in microalgae and that TOR activity modulation is a promising strategy for improving TAG accumulation in microalgae. In the *C. merolae* genome database, CMR018, is denoted as CmTOR with a conserved focal adhesion target (FAT) and FRB, FAT C-terminus (FATC), and kinase domains [19]. Five different FKBP12 homologs were found in the *C. merolae* genome, whereas studies indicated that growth inhibition was not observed even under a high rapamycin concentration [19]. Similar to higher plants, these factors indicate that *C. merolae* may be resistant to rapamycin or have different FKBP amino acid compositions that form a complex with rapamycin. The expression of the *S.* *cerevisiae* FKBP12 protein in *C. merolae* resulted in sensitivity to rapamycin and the resulting strain, F12, was used to study TOR signaling in this alga [19].

Treatment of *C. merolae* F12 with rapamycin indicated the accumulation of lipid droplets as soon as 24 h after treatment; these droplets were not observed in the control culture, which indicates that TOR inactivation is responsible for TAG accumulation in *C. merolae* F12 strain [5]. Additionally, rapamycin treatment resulted in growth inhibition in the F12 strain [37]. A quantitative estimation using GC-FID analysis indicated an almost 8.8-fold higher TAG accumulation after rapamycin treatment than its drug control, DMSO [5]. Compared with the control strain, rapamycin treatment inhibited *C. merolae* F12 growth. As seen in most eukaryotes, under growth inhibition, cells generally accumulate a high amount of energy-reserved compounds such as TAGs. To further verify that TAG accumulation was not caused by growth inhibition in *C. merolae*, our research group conducted a study with the addition of the camptothecin-topoisomerase I inhibiter, a potential growth inhibitor, and found no lipid droplets in the *C. merolae* F12 strain, which confirmed that TAG accumulation under rapamycin treatment was not due to growth inhibition but probably as a result of TOR signaling involvement [5]. Similar to *C. merolae*, the green model alga *C. reinhardtii* also accumulated a high amount of TAGs under TOR inactivation after rapamycin, AZD8055, and Torin 1 treatments [37]. Apart from model microalgae, the photosynthetic protist *Euglena gracilis* and the diatom *Phaeodactylum tricornutum* also accumulated TAGs after rapamycin treatment [38,39]. These studies indicate that the TOR signaling pathway is involved in TAG accumulation, and it is likely that this pathway is conserved among divergent eukaryotic algae.

Induction of TAG accumulation via TOR inactivation is a promising approach compared with other conditions of nutritional depletion such as nitrogen depletion, considering that large-scale nitrogen depletion is practically impossible. Large-scale addition of inhibitors is easy and practical; however, TOR plays a pivotal role in cell growth. Therefore, TOR inhibition leads to cell growth inhibition. Similarly, cell proliferation is inhibited when TAG is accumulated, such as under nitrogen depletion conditions. Furthermore, large-scale addition of rapamycin is expensive. Therefore, to enhance growth and TAG accumulation simultaneously, some alternative methods should be applied. TAG accumulation and cell growth are in a trade-off relationship, and compatibility is one of the most critical issues for improving TAG productivity in microalgae and promoting its future use as a sustainable biofuel.

To solve these issues, we determined how TOR signaling is involved in TAG accumulation in *C. merolae*. Our group performed rapamycin-treated cell transcriptome analysis to understand the underlying molecular mechanisms. The results of transcriptome analysis indicated that after rapamycin treatment, 148 genes were upregulated and 64 genes were downregulated. Among the upregulated genes, 71 genes were upregulated under nitrogen depletion conditions, indicating that both responses partially overlapped [5]. Among the 71 genes, a few genes were also involved in nitrogen assimilation. This indicates that, similar to other eukaryotes, TOR might be involved in regulating nutrients, particularly in nitrogen sensing, in *C. merolae*. Transcriptome analysis revealed that after rapamycin treatment, many genes associated with fatty acid synthesis were downregulated; however, the transcript level of *CMA017C* (the gene number in the *C. merolae* database, *CmGPAT1*), *CMK217C* (*CmGPAT2*), and *CMB069C* (*CmDGAT1*), which were denoted as glycerol-3-phosphate acyltransferase (GPAT), GPAT, and acyl-CoA: diacylglycerol acyltransferase (DGAT), respectively, were upregulated [5].

On the basis of the transcriptome data, our research group tried to confirm the role of CmGPATs (CMA017C and CMK217C) in TAG accumulation in *C. merolae*. The two gene transcripts were upregulated under both nitrogen depletion and TOR inactivation conditions. As localization of enzymes is essential to understand the function of the protein, localization of CmGPAT1 and CmGPAT2 in *C. merolae* was studied. We found that both genes were localized in the ER, indicating that these genes might be involved in TAG accumulation [40]. To further prove our hypothesis, we constructed CmGPAT1 and CmGPAT2 overexpression strains (CmGPAT1ox and CmGPAT2ox, respectively) and evaluated their growth and TAG accumulation characteristics. Results indicated that CmGPAT1ox accumulated a high amount of TAG even under normal growth conditions, whereas TAG content in CmGPAT2ox was similar to that in the control strain [40]. Further quantitative TAG analysis indicated that TAG accumulation in the CmGPAT1ox strain under normal growth conditions was 19 times higher than that in the control strain, which is similar to the amount of TAG accumulated under nitrogen depletion conditions after 72 h. By contrast, in addition to a drastic increase in TAG productivity (>56 fold, 4% dry cell weight of TAG), CmGPAT1ox growth was similar to that of the control strain, which makes this approach more suitable for large-scale TAG production through microalgae [40]. In microalgae, the TAG biosynthesis pathway shares some steps with phospholipid synthesis, an essential component of membrane lipids [41]. Therefore, CmGPAT1 may also be involved in phospholipid biosynthesis in *C. merolae*. However, the CmGPAT1ox strain did not show any significant difference in the mount of phospholipid; therefore, further analysis and research are required to understand this process. Apart from GPAT, DGAT also catalyzes a rate-limiting step for TAG accumulation in various microalgae, such as *C. reinhardtii*, *N. oceanica*, and *Neochloris oleoabundans* [42,43,44]. However, in *C. merolae*, CmDGAT1 overexpression, whose transcription was increased because of TOR inactivation [5], did not show any improvement in TAG production under normal growth conditions [40]. This indicates that the reaction catalyzed by ER-localized GPAT was a rate-limiting step in the TAG biosynthesis pathway in *C. merolae*. As TOR and ER-localized GPAT are widely conserved proteins in eukaryotes, the regulatory mechanism of TOR contributes to TAG accumulation by upregulating ER-localized GPAT expression, which might be conserved among microalgae. Furthermore, identifying TOR-regulated transcription factors involved in the regulation of CmGPAT1 expression will enhance our understanding of TAG synthesis and accumulation pathway in *C. merolae*. This knowledge will lead to the production of renewable biomass and biofuels from microalgae without compromising biomass production, which is an important step for large-scale applications (Figure 1).

During preparation of this article, our group identified and studied 14 transcription factors, which might involve in TAG accumulation in *C. merolae* based on transcriptome and phosphoproteome data [5,6]. Among these 14 transcription factors, it was revealed that BRD1, HSF1, MYB3, and MYB4 are involved in LD formation and TAG accumulation. Further RNA-seq analyses using each overexpression strain revealed that the four transcription factors control LD formation and TAG accumulation through changing the expression of gene encoding ER-localized lysophosphatidic acid acyltransferase 1 (LPAT1) [45]. Additionally, overexpression of LPAT1 of *C. merolae* (CmLPAT1) resulted in TAG accumulation under normal growth conditions being 3.3 times higher than that in the control strain, suggesting that the step catalyzed by CmLPAT1 is another rate-limiting step in TAG biosynthesis in *C. merolae*.

Apart from genes involved in fatty acid and TAG syntheses, genes involved in degradation or consumptions of fatty acid and TAG are also important targets to improve the TAGs content in microalgae. In this regard, for *C. merolae*, the previous DNA microarray analysis obtained under 24 h after -N indicates that transcripts of β-oxidation are related, and TAG lipase genes are not upregulated, and some of them were downregulated [5]. Under the TOR-inactivation condition with rapamycin, transcripts of genes encoding acyl-CoA oxidase, which is involved in β-oxidation, and one TAG lipase were increased [5]. So, disruption or repression of these genes would be potential targets to further increase TAG contents under the TOR-inactivation condition.

## 4. Genetic Engineering of the Fatty Acid Transporter to Improve Biofuel Production

In various life forms, such as bacteria, fungi, and humans, fatty acids are synthesized using specialized enzymes such as ACCase. In plants and algae, different forms of ACCase are located in plastids and cytosol, which are responsible for de novo fatty acid synthesis [46]. Among various metabolic engineering strategies, modifying or improving lipid trafficking is a promising method to enhance TAG content in cells. Reportedly, overexpression of AtFAX1, which is involved in chloroplast fatty acid export in the model plant *A. thaliana*, enhanced TAG accumulation in cells [47]. A homology search in the *C. merolae* genome database (http://czon.jp, accessed on 28 April 2021) identified one AtFAX1 similar homolog, CMA070C, which is denoted as a hypothetical protein. CMA070C (hereafter, CmFAX1) showed 27 and 31% similarity with human TMEM14A and TMEM14C, respectively, which belong to the Tmemb_14 superfamily [46], and showed similarity with land plant FAX proteins [48]. Additionally, CmFAX1 contains a transit peptide at its amino-terminal and shows 25% identity with AtFAX1. Fat1 is a similar protein in yeast, which is a transmembrane transport protein involved in the influx of exogenous fatty acids [49]. Wild-type yeast cannot survive in a media supplemented with exogenous fatty acids, such as α-linolenic acid, whereas the Fat1 mutant is insensitive to this addition [49]. Functional complementation of CmFAX1 in yeast lacking Fat1 indicates its inability to grow in media supplemented with α-linolenic acid and that it is the fatty acid exporter for *C. merolae* [48]. Further immune fluorescence-based localization studies indicate that CmFAX1 is localized in the chloroplast periphery. Taken together, this suggests that CmFAX1 plays a role in fatty acid transport from chloroplast to the cytosol in *C. merolae* [48].

In photosynthetic organisms, chloroplast is generally the main site for fatty acid synthesis, and it transports fatty acid to the ER. Under various stress conditions, de novo fatty acid are synthesized in the ER, whereas under the normal conditions, FAX1 overexpression in *C. merolae* resulted in the accumulation of almost 2.4-fold higher TAG production, which proves the role of CmFAX1 in fatty acid export in this alga (Figure 1). Further analysis revealed that contents of short-chain free fatty acids (FFAs), such as 14:0 and 14:1, in cells might be attributable to over-transport in the CmFAX1ox strain. Additionally, the CmFAX1 null mutant showed an almost 2.5-fold increase in FFA content [48]. Fatty acid analysis of the CmFAX1 null mutant indicates the accumulation of short-chain FAs containing TAGs, such as 14:0 or 14:1, and very-long-chain FAs containing TAGs, such as 20:0. This might be due to the overproduction of the CmFAX1 protein in the CmFAX1ox strain, which might transport incomplete elongated FAs from chloroplast, and an increase in 20:0 FA, which is generally synthesized in the ER surface. One of the interesting phenotypic characteristics of CmFAX1ox strain is its paler green color compared with the control strain. Chlorophyll-a analysis indicates that the CmFAX1ox strain has a lower Chl-a content, which caused it to be paler. This reduction in pigments might be due to a change in the fatty acid composition of the chloroplasts due to the export of fatty acids to the ER. However, CmFAX1ox strain grow rapidly compared with the control strain. One of the possible explanations for this higher growth rate is the increase in light penetration ability because of the low Chl-a content, even in a high-density culture. This is a very useful characteristic for large-scale cultivation and biofuel production [48].

## 5. Metabolic Engineering to Improve the Starch Content in *C. merolae*

Microalgal carbohydrates are an excellent bioresource for the production of bioethanol and various specialty chemicals, such as methyl lactate and methyl levulinate [50,51]. Usually, the accumulation of energy-reserved compounds, such as lipids and starch, occurs under unfavorable growth conditions, such as nitrogen depletion. However, most studies have focused only on the utilization of the TAG component for biodiesel production. Leftover biomass after lipid extraction, which is rich in carbohydrates, remained unutilized. Microalgal carbohydrates are important compounds that can be easily converted into industrially important chemicals such as lactic acid using homogeneous catalysts [51]. After TAGs, a major carbon-reserved molecule in *C. merolae* is floridean starch (hereafter, starch), of which contents increase together with TAGs after -N [52]. In higher plants and green algae, starch is accumulated in the chloroplast of cells, whereas in *C. merolae*, starch accumulation is generally observed in the cytoplasmic area [53,54]. Additionally, *C. merolae* uses uridine diphosphate-glucose instead of adenosine diphosphate (ADP) as a precursor for starch synthesis [55]. Similar to higher plants, *C. merolae* also accumulate starch during light conditions and degrade the carbon source in the dark for respiration [52,56]. Understanding the mechanism or components that regulate starch synthesis and degradation will help us improve the accumulation of such compounds in cells. Starch was seen to accumulate in *C. merolae* cells very early (24 h after nitrogen depletion) and remained for up to 120 h after nitrogen depletion, which was visualized through KI staining [52]. This result indicates that starch is not degraded to form a lipid body in *C. merolae,* which indicates this for TAG synthesis carbon might obtain from other carbon reserves of cells like amino acid or membrane lipid [52].

Understanding the molecular mechanism behind starch accumulation is important for utilizing microalgal starch for bioethanol and other production methods for valuable compounds. In *C. merolae*, gene expression analysis under nitrogen depletion and TOR inactivation conditions, which lead to starch accumulation, indicated no transcript-level changes in the key genes involved in starch accumulation, such as glycogenin, starch synthase, the de-branching enzyme, and glycogen phosphorylase, etc. [5,52]. This indicates that regulators for starch metabolism may act at the posttranscriptional levels. In *C. merolae,* a significant amount of starch accumulated after 12 h of rapamycin treatment, and an almost 10-fold higher starch accumulation was observed after 48 h of rapamycin treatment, which suggests that TOR plays a role in the regulating starch accumulation in *C. merolae* [6]. Additionally, the starch content did not differ because of rapamycin treatment, and rapamycin treatment, along with nitrogen depletion, indicates that TOR is a major pathway, at least under nitrogen depletion, which regulates starch accumulation in cells [6]. As mentioned above, starch metabolism might be regulated at the posttranscriptional levels in *C. merolae* [5]. As TOR is a kinase, it is possible that TOR regulates starch metabolism in *C. merolae* through the phosphorylation of target proteins.

**Figure 2:**
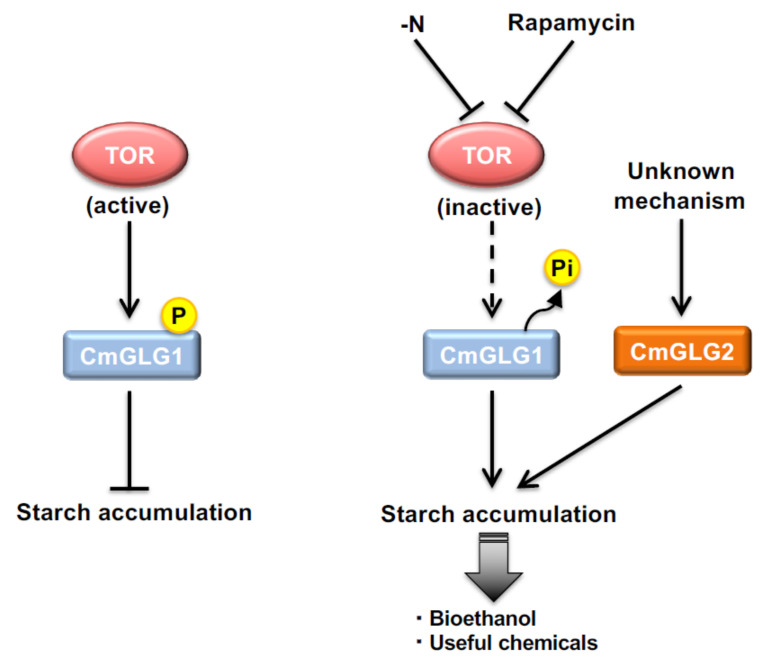
The metabolic engineering strategies to improve starch content in *Cyanidioschyzon merolae*. In this alga, TOR is also the checkpoint kinase for the starch accumulation in addition to the TAG synthesis. Under the TOR inactivation by rapamycin or nitrogen depletion conditions, serine 613 residue of CmGLG1 is de-phosphorylated, and the de-phosphorylated form of CmGLG1 triggers the accumulation of a high amount of starch in the cells. Another protein, CmGLG2, which is also similar to glycogenin, is also involved in the accumulation of starch in this alga; however, the mechanism behind such accumulation is not known yet. Further studies might help to reveal such mechanism and sustainable use of *C. merolae* biomass for the production of bioethanol and other valuable chemicals.

To verify this possibility, our group conducted phosphoproteomic analyses by treating *C. merolae* with rapamycin for 2, 8, and 24 h and compared the changes in phosphorylation with the drug control, DMSO [6]. The results indicated that TOR regulates various metabolic processes in cells, such as starch metabolism and photosynthesis. Among the various proteins, CMK020C (CmGLG1), which is similar to glycogenin, is crucial for starch metabolism in *C. merolae*. Glycogenin is a key protein that initiates starch/glycogen synthesis in yeast and mammals through self-glycosylation by creating a short primer of glycogen, and further elongation occurs through the action of starch/glycogen synthetase [57]. Tyr194 residue in glycogenin protein is essential for the self-glycosylation reaction, and this amino acid is conserved in CmGLG1 [6]. However, glucosylation is not the only mechanism that regulates glycogenin activity; a mutation in Tyr194 with phenylalanine or threonine did not affect the glycogen initiation process, which indicates that, aside from glycosylation, other mechanisms such as phosphorylation might regulate the activity of glycogenin, as the addition of 10 mM ATP reduced the activity of recombinant glycogenin by 45% [58,59]. Overexpression of CmGLG1 in *C. merolae* yields 4.7-fold high accumulation in starch compared with that of the control strain, which indicates that CmGLG1 is involved in starch accumulation in *C. merolae* [6]. Rapamycin-treated phosphoproteome indicates that phosphorylation of Ser613 residue of CmGLG1 decreased after rapamycin treatment; subsequently, 60% reduction in starch accumulation was observed when Ser613 residue was changed with aspartic acid [6]. These results indicate that under unfavorable conditions such as nitrogen depletion, TOR inactivation results in dephosphorylation of CmGLG1 and subsequently a high accumulation of starch content in cells (Figure 2).

In the *C. merolae* genome, CmGLG1, CMG174C, CMR358C, and CMJ262C have shown similarity with glycogenin [11,12,60,61]. Among these four proteins, CMR358C and CMJ262C can synthesize polyglucans and are involved in mitochondrial and chloroplast division, respectively, and are denoted as MDR1 and PDR1 [60,61]. All the glycogenin-type proteins contain a glucosyltransferase domain, which means they can transfer sugar. Phylogenetic analysis indicated that CMG174C was a cluster of yeast and rabbit glycogenin, which introduced the possibility that, similar to yeast and mammals, *C. merolae* contains two glycogenin proteins [62]. CmGLG1 formed a cluster with the plant glycogenin-type protein, whereas MDR1 and PDR1 were in different clusters, indicating that these proteins might not be involved in starch accumulation but rather only in organelle division. Overexpression of CMG174C (hereafter denoted as CmGLG2) under a strong APCC promoter results in almost two-fold higher starch content than that in the control strain [62], which indicates that CmGLG2 might be responsible for starch accumulation in *C. merolae*. However, the regulatory mechanism through which CmGLG2 accumulates starch remains unknown. Similar to CmGLG1, the mRNA level of CmGLG2 was also not affected by nitrogen depletion or rapamycin treatment, which indicates that regulation might be at a posttranscriptional level that is similar to CmGLG1 [5]. However, CmGLG2 was not detected in the phosphoproteomic study conducted under TOR inactivation [6], which indicates the involvement of some other posttranslational mechanism that was missed under our experimental phosphoproteomic analysis setup. Further analysis is required to understand how the interrelationship of CmGLG1 and CmGLG2 is involved in starch accumulation and the molecular mechanism underlying the starch accumulation via CmGLG2 overexpression (Figure 2).

## 6. Conclusions and Prospects

Production of renewable fuel and other valuable chemicals from microalgal biomass is a promising and green approach; however, because of various regulatory issues, the large-scale production of genetically modified algae is not advisable. In this regard, *C. merolae* is one of the most suitable algae for such applications. Additionally, various target genes, transcription factors, and signaling pathways have been identified through various analyses, such as transcriptomics and phosphoproteomics, which might be responsible for the regulation of energy-reserved compounds in cells [5,6]. Identification of the upstream signaling pathway, tight regulation and distribution of ATP, and reducing power are among the various suitable alternative genetic engineering strategies that may be employed for the production of suitable algal biomass. However, numerous genetically engineered strains failed to produce a high amount of biomass along with TAGs/starch accumulation in cells because of the unbalanced regulation flux of carbon and energy in such strains. In *C. merolae*, the TAG and starch synthesis pathway is almost similar to higher plants and other green algae, except for a few differences. However, the full molecular mechanism has not been elucidated, and there are various knowledge gaps, such as which signaling pathway(s) as well as TOR is involved in the accumulation of TAGs and starch, and which enzyme(s) and its cellular localization are responsible for lipid droplet biogenesis or lipid trafficking. Among the different signaling pathways, TOR is an ancient kinase that regulates growth and metabolic status in yeast and mammals. A recent study indicated that this kinase is also conserved in plant lineages, including microalgae [63,64]. One of the major research aims in our lab is to understand how TOR regulates the accumulation of energy-reserved compounds in model alga *C. merolae* [5,6,37,40]. Among the various studies, rapamycin-induced transcriptome indicates a massive rearrangement of the genes that are involved in various physiological processes, such as photosynthesis and the accumulation of TAGs in *C. merolae*. In this gene expression analysis, one of the identified targets was CmGPAT1. Overexpression of CmGPAT1 resulted in a more than 56-fold higher TAG productivity compared with that of the control strain; this indicates a novel step in TAG biosynthesis in microalgae, which is regulated by TOR signaling. In model green alga *Chlamydomonas*, generally, overexpression of DGAT proteins, which catalyzes the final step in the TAG biosynthetic pathway, resulted in TAG accumulation. This indicates that our understanding of the biosynthetic pathway for TAG in the microalgae is not yet clear, and many unknown mechanisms are involved in the process. In future, analysis of the interrelationship between DGAT, GPAT, and TOR will lead to a greater understanding underlying the molecular mechanism of TAG accumulation in algae and its commercial application as biofuel.

Microalgal starch is an essential precursor for the synthesis of biofuel and other industrially important chemicals. If we compare various biomasses for the supply of starch, microalgae are among the most suitable biomasses, as they contain a very low amount of hemicellulose and do not contain lignin in their cell walls [64]. Lignin is a very complex biopolymer that requires harsh pretreatment. During pretreatment, various inhibitory compounds are also produced, which will affect the growth of fermentative microorganisms. Similar to TAGs, starch also accumulates under TOR inactivation conditions in various microalgae; however, the molecular mechanism behind this is not clear. In *C. merolae,* phosphorylation CmGLG1 of Ser613 changes after rapamycin treatment. Overexpression of CmGLG1 enhances starch production by almost 4.7-fold compared with that in the control, which indicates a promising strategy for improving the starch content in microalgae. Further studies are required to better understand the metabolic regulation of starch and TAG accumulation in *C. merolae* and how the central regulator TOR is involved in such processes. Identification of the underlying mechanism will help generate a sustainable and green biomass from the microalgae, which will help produce renewable fuel and other valuable chemicals from microalgae as a bioresource that can save the environment and economy.

## Data Availability

Not applicable.
